# Bolstering the Evidence Base for Integrating Abortion and HIV Care: A Literature Review

**DOI:** 10.1155/2012/802389

**Published:** 2012-12-18

**Authors:** Ruth Manski, Amanda Dennis, Kelly Blanchard, Naomi Lince, Dan Grossman

**Affiliations:** Ibis Reproductive Health, 17 Dunster Street, Suite 201, Cambridge, MA 02138, USA

## Abstract

HIV-positive women have abortions at similar rates to their HIV-negative counterparts, yet little is known about clinical outcomes of abortion for HIV-positive women or the best practices for abortion provision. To fill that gap, we conducted a literature review of clinical outcomes of surgical and medication abortion among HIV-positive women. We identified three studies on clinical outcomes of surgical abortion among HIV-positive women; none showed significant differences in infectious complications by HIV status. A review of seven articles on similar gynecological procedures found no differences in complications by HIV status. No studies evaluated medication abortion among HIV-positive women. However, we did find that previously expressed concerns regarding blood loss and vomiting related to medication abortion for HIV-positive women are unwarranted based on our review of data showing that significant blood loss and vomiting are rare and short lived among women. We conclude that although there is limited research that addresses clinical outcomes of abortion for HIV-positive women, existing data suggest that medication and surgical abortion are safe and appropriate. Sexual and reproductive health and HIV integration efforts must include both options to prevent maternal mortality and morbidity and to ensure that HIV-positive women and women at risk of HIV can make informed reproductive decisions.

## 1. Introduction

More than half of all people living with HIV worldwide are women, and most of them live in low- and middle-income countries [1]. Facilitating access to comprehensive sexual and reproductive health (SRH) services for women is currently the focus of many global health interventions, in part as a result of efforts to achieve Millennium Development Goal 5b (achieving universal access to SRH).  However, few of these interventions focus on the specific challenges of facilitating access to SRH services for HIV-positive women. Fewer still focus on ensuring access to safe abortion for HIV-positive women. 

HIV-positive women, like all women, require the right to access safe abortion services to prevent maternal mortality and morbidity and to be able to exercise their reproductive rights. Documented rates of unintended pregnancies among HIV-positive women highlight the importance of access to safe abortion for this population, for example, studies have shown that between 51% and 91% of pregnancies among HIV-positive women are unintended [2]. Furthermore, studies show that HIV-positive women have abortions at similar rates to their HIV-negative counterparts and seek abortions for many of the same reasons other women do, such as economic constraints or lack of partner support [3–9]. However, HIV-positive women face particular challenges when trying to access abortion services. 

In addition to issues that affect women regardless of HIV-status, such as lack of information about abortion options, and financial barriers to abortion care, abortion is also legally restricted in many low- and middle-income countries where HIV is prevalent—limiting positive and negative women's access to information and services. For example, in Southern Africa, where HIV rates are the highest in the world, abortion is only available without legal restriction in South Africa [10]. Furthermore, the confluence of stigma surrounding HIV/AIDS and abortion may make it even more difficult for HIV-positive women to access abortion services. Several studies have documented that HIV-positive women experience discrimination by health care providers based on their reproductive decisions, with some women facing pressure to carry a child to term and others to have an abortion [11–13]. Furthermore, unique clinical questions, such as concerns about transmitting HIV during pregnancy and fears about how a pregnancy will affect a woman's health, may impact HIV-positive women's abortion-related decision making [11]. Clinical concerns may also affect providers' or policymakers' decisions to make certain types of abortion services available to HIV-positive women. In particular, some authors have suggested that medication abortion may not be appropriate for HIV-positive women because they are at higher risk for anemia and that vomiting from medication abortion drugs may reduce the efficacy of HIV treatment drugs [12, 14].

The separation of abortion services from both SRH services and HIV services may present further obstacles to HIV-positive women's access to safe abortion care [15]. Efforts to integrate SRH and HIV services have become a priority in many health care settings, due to the potential benefits of integrating health care services, including increasing access to services and improving the quality of care [16]. However, integration research and programs have largely focused on including family planning and prevention and management of sexually transmitted diseases in HIV-focused services (or vice versa), but generally do not address safe abortion for HIV-positive or -negative women. Some models that could be used to integrate abortion and HIV services include providing abortion care in the same clinic where women receive HIV care and treatment or including referrals from one service to another. In settings where abortion is legally restricted, harm reduction counseling about safe and effective misoprostol regimens could be integrated into the HIV care setting.

Despite the documented need for abortion among HIV-positive women [3–8], there has been little research that addresses clinical outcomes of surgical and medication abortion for HIV-positive women or the best practices for abortion service provision and care. This paper reviews the existing data on clinical outcomes of surgical and medication abortion among HIV-positive women, with a focus on complication rates and adverse events. Given the limited amount of data available on this issue, we have selected a small group of gynecological procedures that are of similar or greater invasiveness when compared to abortion and review the literature for those procedures conducted among HIV-positive women. We, then, summarize data on blood loss and vomiting experienced by some women undergoing medication abortion to address concerns about the differential impact of these side effects on HIV-positive women. 

## 2. Methods

We conducted two separate literature reviews which were supplemented with focused searches to identify additional literature. For the first review, from August to October 2011, we searched PubMed for studies that reported on clinical outcomes of surgical and medication abortion among HIV-positive women using the keywords “abortion” or “termination of pregnancy” and “HIV.” Only original research studies published in English with female human subjects were eligible for inclusion. We scanned the titles, abstracts, and citations of all articles and removed any that were irrelevant, such as articles focusing on the reproductive rights of HIV-positive women or the characteristics of HIV-positive women seeking abortion care. 

For the second review, focusing on gynecological procedures deemed to be similar to abortion as explained previously, from March to May 2012, we searched PubMed using the following search terms: “HIV” and “LEEP” (loop electrosurgical excision procedure), “laparoscopy,” “cone biopsy,” “fibroid treatment,” “endometrial biopsy,” “endometriosis management,” “myomectomy,” or “uterine cervical neoplasms diagnosis, surgery, or therapy [MESH].” (Medical Subject Headings (MeSH) is the National Library of Medicine controlled vocabulary thesaurus used for indexing articles for PubMed). We did not find any studies using the search terms “HIV” and “fibroid treatment,” “endometriosis management,” or “myomectomy.” As in our first review, we considered original research studies published in English with female human subjects eligible for inclusion. We scanned the titles, abstracts, and citations of all articles and removed any that did not discuss complications among HIV-positive women. 

For the third and fourth searches, to address concerns expressed regarding medication abortion for HIV-positive women, in October 2011, we searched PubMed for articles on (1) changes in hemoglobin levels due to blood loss from medication abortion where anemia is prevalent (discussed below) and (2) the frequency and duration of vomiting due to medication abortion drugs. Our aim was not to conduct an exhaustive review of this expansive body of the literature, but rather to identify key articles and identify the severity and frequency of blood loss and vomiting among women choosing medication abortion. 

To identify articles on blood loss, we limited the search to India, because there is a high prevalence of anemia among Indian pregnant women [17, 18], and a large number of medication abortion studies have been conducted in India. Our search terms included “medical abortion” or “medication abortion” and “India;” these results were then narrowed down by searching within the text of each article for the keywords “anemia” or “hemoglobin.” Only original research studies published in English with female human subjects were considered eligible for inclusion. We scanned the titles, abstracts, and citations of all articles and removed any that did not include “anemia” or “hemoglobin” as a keyword.

To identify articles on vomiting associated with medication abortion drugs, we used the search terms “medical abortion” or “medication abortion” and “side effects.” The search results were then narrowed down by searching within the text of each article for the keyword “vomit.” Only English studies with female human subjects were eligible for inclusion. We reviewed both original research articles and literature reviews. We scanned the titles, abstracts, and citations of all articles and removed any that did not include information on the frequency and/or duration of vomiting. We ultimately focused on the findings from a literature review that summarized data on vomiting from medication abortion drugs. None of the searches were limited to specific dates of publication.

## 3. Results

Our first literature review on clinical outcomes of surgical and medication abortion identified 559 articles. No articles discussed clinical outcomes of medication abortion among HIV-positive women. Three articles that discussed clinical outcomes of surgical abortion among HIV-positive women met our criteria and were included in our review. 

### 3.1. Surgical Abortion

Detailed information on each of the three articles that examined clinical outcomes of surgical abortion among HIV-positive women is included in Table 1. The first study, conducted in urban Uganda, explored whether HIV was a risk factor for postabortion endometritis-myometritis (PAEM) [19]. PAEM was defined as an incomplete abortion (admitted induced or spontaneous abortion) with intrauterine infection. Of women with PAEM, 32.7% were HIV-positive, and of women without PAEM, 36.5% were HIV-positive. The difference was not statistically significant. 

The second study examined morbidity associated with curettage for abortion among HIV-positive and -negative women in a public hospital in Texas, USA [20]. Women with septic abortions were excluded from the study, and only a minority of subjects had AIDS. There were higher numbers of anesthesia-related complications and instances of retained placenta among HIV-positive women. However, the numbers were small (two and three, resp.), and the authors posit that instances of retained placenta were unlikely related to HIV-status as there is no known biologic susceptibility to retained placental tissue among HIV-positive women. Three percent of HIV-positive women and 1% of HIV-negative women experienced infectious complications; the difference between groups was not significant. 

In the third study, the authors compared the risks of postoperative morbidity among HIV-positive and -negative women undergoing six categories of surgical procedures, including curettage [21]. Among patients experiencing major and minor complications after curettage (e.g., fever requiring antibiotics, additional surgical procedures, anemia, urinary tract infection, development of endometriosis and lochiostasis after obstetric procedures, and disseminated intravascular coagulation), 9.7% were HIV-positive and 1.4% were HIV-negative. However, the point estimates must be viewed with caution due to the small number of women in the sample who experienced relevant complications (7 HIV-positive women and 1 HIV-negative woman), the width of the confidence intervals (0.92–63.84), and the lack of statistical significance; it was also impossible to separate out minor from major complications based on the published data. 

### 3.2. Other Gynecological Procedures

Due to the lack of data specifically addressing clinical outcomes of surgical abortion among HIV-positive women, we reviewed the literature on clinical outcomes of gynecological procedures deemed to be of similar or more invasiveness to abortion. We identified 144 articles. Seven articles, which provide clinical outcomes after LEEP and laparoscopic sterilization among HIV-positive women, met our inclusion criteria. 

We summarize details of each of the seven studies in Table 2. We found one article on laparoscopic sterilization for HIV-positive women in Thailand [22]. The authors found that laparoscopic sterilization was safe. No study participants experienced immediate or subsequent surgical complications, though the authors did not provide detail regarding what complications were measured. Six studies examined complications among HIV-positive women undergoing LEEP. One study, conducted in Kenya, measured women's self reports of the presence and severity of complications, including bleeding, discharge, and pain [23]. No participants reported severe symptoms, and only 1% (*n* = 1) reported moderate symptoms. The other five studies, conducted in Thailand and Zambia, measured postoperative and intraoperative bleeding-related complications including hemorrhage and rates of postoperative infection among HIV-positive and -negative women undergoing LEEP [24–28]. There were no significant differences in complication rates based on HIV-status. 

### 3.3. Medication Abortion

To address concerns raised in the literature regarding risks of blood loss and vomiting related to medication abortion for HIV-positive women, we conducted a review of the existing data on blood loss with medication abortion in India—a setting with a high prevalence of anemia. Our literature search identified 39 articles, five of which were included in our review. These are summarized in Table 3. While no studies were identified that specifically measured outcomes of medication abortion among women with anemia, all of the studies were conducted in India where the majority of pregnant women have anemia [17, 18]. Three of these articles reported findings from multicountry studies conducted in India, Cuba, and China [29–31]; findings reported in the text and Table 3 focus only on the India data. 

Clinically significant changes in hemoglobin levels due to blood loss from medication abortion were rare in all studies. Mean changes in hemoglobin levels between study enrollment and the follow-up visit (12–14 days later) ranged from 0.1 to 0.29 gm/dL, and the percent of women whose hemoglobin levels dropped 2 gm/dL ranged from 1.2 to 4% [29–33]. In one study, one woman required a blood transfusion [30]. 

Our review of vomiting from medication abortion identified 147 articles, of which 66 were relevant. One literature review of nine studies comparing medication abortion practiced in homes and clinics using the evidence-based medication abortion regimen of 200 mg mifepristone and 400 *μ*g misoprostol orally provided vomiting data for four studies conducted in Albania, Nepal, Turkey, and Vietnam [34]. Reported rates of vomiting ranged from 11.5 to 33.7%, and vomiting lasted an average of less than one day. Other relevant articles identified in our search reported similar rates and durations of vomiting among women undergoing medication abortion, although there is variation based on the medication abortion drug regimen and other study variables.

## 4. Discussion 

Our review identified no studies that assessed clinical outcomes of medication abortion and three studies that assessed clinical outcomes of surgical abortion among HIV-positive women. Despite the few studies, existing data show that adverse outcomes of surgical abortion in HIV-positive women are low and occur at similar rates to HIV-negative women. Similarly, the literature on LEEP and laparoscopic sterilization, two procedures that are of similar or more invasiveness to abortion, shows that adverse events when these procedures are performed among HIV-positive women are uncommon and not significantly different according to HIV-status. In addition to this data, clinical guidelines on the management of cervical intraepithelial neoplasia (CIN) specify that recommended treatment for HIV-positive women is the same as for women in the general population [35]. In the absence of other data, these findings can be used to make inferences about the safety of surgical abortion for HIV-positive women. 

Data also suggests that medication abortion is safe for HIV-positive women. First, data reviewed on blood loss from medication abortion in India, where there is a high prevalence of anemia among pregnant women [17, 18], shows that significant changes in hemoglobin levels are infrequent. Additionally, because the majority of women do not experience vomiting associated with use of medication abortion drugs, and, for those that do, the average duration is less than one day, the impact of vomiting on HIV treatment drugs is likely short lived. It is also important to note that vomiting is common during pregnancy and would likely last much longer than one day. To avoid any potential (although unlikely) reductions in HIV treatment drug efficacy, women could repeat a dose if vomiting occurs less than one hour after taking HIV treatment drugs and/or begin the medication abortion drug regimen at least one hour after HIV treatment drugs [36]. Further documentation of the safety of medication abortion for HIV-positive women comes from medication abortion studies in HIV-prevalent countries. While there is a need for more research, clinical outcomes of medication abortion from countries with a high prevalence of HIV, like South Africa, are similar to reports from countries with a lower HIV prevalence [37]. 

There are several limitations of this literature review. First, the outcome of interest was not consistently defined across surgical abortion studies, making it difficult to compare findings. In addition, the varying legal contexts and surgical methods of abortion, as well as inclusion or exclusion of women with septic abortions, limit the ability to make generalizations about clinical outcomes of abortion for HIV-positive women. In the absence of specific data on surgical and medication abortion among HIV-positive women, our review on clinical outcomes of LEEP and laparoscopic sterilization as well as blood loss and vomiting from medication abortion may provide helpful insight regarding abortion care for HIV-positive women. However, we acknowledge that there are clinical differences when considering abortion and LEEP or laparoscopic sterilization, and the data presented here on blood loss and vomiting from medication abortion are not focused on HIV-positive women. 

Further research is needed which evaluates the complications of safe abortion among HIV-positive and -negative women. Research documenting clinical experience and acceptability of medication and surgical abortion among HIV-positive women would be useful to inform discussions about standards of care. Furthermore, research examining HIV-positive women's choice of method and the impact of particular methods on HIV-treatment would strengthen recommendations and identify best practices for abortion care for HIV-positive women. Until such data are available, we are confident that our review provides a strong argument for offering HIV-positive women both surgical and medication abortion where available; offering women the choice of methods would be ideal.

Efforts aimed at integrating abortion services into SRH and HIV services are critical to protecting and promoting the reproductive health and rights of HIV-positive women. SRH and HIV providers not only need to have the clinical skills and equipment to perform abortions, but also the capacity to provide nonjudgmental counseling to HIV-positive women on their reproductive rights and choices to meet women's sexual and reproductive health needs. In addition, expanding access to and providing HIV-positive women who do not wish to become pregnant with the full range of contraceptive options is a critical component of SRH/HIV integration and could support women in achieving their reproductive goals. 

We recognize that there are policy- and service-level challenges to integrating SRH and HIV services, such as legal and funding constraints, lack of national guidelines on integration, and staffing shortages [38, 39]. In settings where abortion is legally restricted, women could be provided with harm reduction counseling about safe and effective misoprostol regimens, which has been shown to decrease complications from unsafe abortion [40], or, in other cases, women with HIV might be eligible for legal abortion if continuing the pregnancy would jeopardize their health. Further, advocates for women's health must hold accountable programs and policies that do not address women's comprehensive SRH needs, including HIV and safe abortion care. The International HIV/AIDS Alliance names abortion as a key HIV and SRH intervention and outlines strategies for how to integrate safe abortion and postabortion care into HIV services [41]. Advocates for HIV-positive women should build on these recommendations and the momentum generated by the 2011 report from the United Nations Special Rapporteur on the Right to Health that calls for the removal of legal barriers to access to abortion for all women to improve women's health worldwide [42].

## 5. Conclusion

Based on the existing data on clinical outcomes of abortion and other gynecological procedures for HIV-positive women, surgical and medication abortion appear to be both safe and appropriate options for HIV-positive women. Improving access to safe abortion services has been shown to lead to significant reductions in maternal mortality and morbidity. Efforts to integrate SRH and HIV services should be expanded to incorporate information about safe abortion and referrals, and/or provision of safe abortion services, and consider the specific needs of HIV-positive women. 

## Figures and Tables

**Table 1:** Studies on surgical abortion among HIV-positive women.

Authors	Study type	Setting	Population	Number	Findings
Grubert et al. (2002) [21]	Retrospective	Germany, specialized outpatient clinic	HIV-positive and -negative women undergoing curettage	72	9.7% (*n* = 7) of HIV-positive women and 1.4% (*n* = 1) of HIV-negative women experienced complications; difference was not statistically significant (*P* = 0.063; OR, 7.65; 95% CI, 0.92–63.84)
Okong et al. (2002) [19]	Prospective	Uganda, gynecological ward at urban hospital	HIV-positive and -negative women with and without postabortion endometritis-myometritis (PAEM)	158	32.7% (*n* = 17) of women with PAEM were HIV-positive, and 36.5% (*n* = 38) of women without PAEM were HIV-positive; difference was not statistically significant (OR, 0.84; 95% CI 0.39–1.80)
Stuart et al. (2004) [20]	Retrospective	Texas, USA, public hospital	HIV-positive and -negative women undergoing curettage for abortion	284	3% (*n* = 2) of HIV-positive women and 1% (*n* = 3) of HIV-negative women experienced infectious complications; difference was not statistically significant (*P* = 0.435). 3% (*n* = 2) of HIV-positive women and no HIV-negative women experienced anesthetic complications; 4% (*n* = 3) of HIV-positive women and 0% (*n* = 1) of HIV-negative women experienced retained placenta.

**Table 2:** Studies on LEEP and laparoscopic sterilization in HIV-positive women.

Authors	Study type	Setting	Population	Number	Findings
Laparoscopic sterilization					

Intaraprasert et al. (1996) [22]	Retrospective	Thailand, university hospital	HIV-positive women undergoing laparoscopic sterilization	18	No immediate or subsequent surgical complications

LEEP					

Kietpeerakool et al. (2009) [26]	Prospective	Thailand, university hospital	HIV-positive and -negative women undergoing LEEP	789	HIV infection was not significantly associated with LEEP complications (OR, 0.41; 95% CI, 0.15–1.15)
Kietpeerakool et al. (2006) [25]	Prospective	Thailand, university hospital	Women with abnormal cervical cytology undergoing LEEP	206	HIV was not an independent risk factor for LEEP complications (*P* = 0.49)
Kietpeerakool et al. (2006) [24]	Retrospective	Thailand, university hospital	HIV-positive and -negative women undergoing LEEP for CIN	120	HIV was not significantly associated with LEEP complications (*P* = 0.24)
Pfaendler et al. (2008) [27]	Prospective	Zambia, primary care clinics and tertiary hospital	Women in a screen and treat cervical cancer prevention program undergoing LEEP	748	Number of women experiencing complications was not large enough to compare significant differences in complications based on HIV-status; complication rates were low in both groups
Sutthichon and Kietpeerakool (2009) [28]	Retrospective	Thailand, university hospital	Women undergoing their first LEEP	857	HIV status was not a significant predictor of perioperative complications
Woo et al. (2011) [23]	Prospective	Kenya, clinics	HIV-positive women who returned for a 4-week followup after LEEP	180	No participants reported severe symptoms; 1% (*n* = 1) reported moderate symptoms; 99% (*n* = 179) reported very mild to mild symptoms

**Table 3:** Studies summarizing data on blood loss due to medication abortion in India.

Authors	Study type	Setting	Population	Number	Medication abortion regimen	Findings
Coyaji et al. (2002) [33]	Prospective	India, urban and rural hospitals	Pregnant women with gestations of ≤63 days in the urban sites and ≤56 days in the rural site	900	600 mg mifepristone and 400 *μ*g misoprostol; oral	Mean change in hemoglobin levels 0.1–0.2 gm/dL
Elul et al. (1999) [31]	Retrospective	India, urban hospital	Pregnant women with amenorrhea ≤56 days	250	600 mg mifepristone and 400 *μ*g misoprostol; oral	Mean change in hemoglobin levels −0.29 gm/dL
Harper et al. (1998) [29]	Prospective	India, urban hospital	Pregnant women with amenorrhea ≤56 days	250	600 mg mifepristone and 400 *μ*g misoprostol; oral	4% of women experienced drop in hemoglobin levels >2 gm/dL
Mundle et al. (2007) [32]	Prospective	India, primary health care center	Pregnant women with amenorrhea ≤56 days	149	200 mg mifepristone and 400 *μ*g misoprostol; sublingual	Median change in hemoglobin levels 0.1 gm/dL; no serious complications
Winikoff et al. (1997) [30]	Prospective	India, urban hospital	Pregnant women with amenorrhea ≤56 days	250	600 mg mifepristone and 400 *μ*g misoprostol; oral	Mean change in hemoglobin levels −0.29 gm/dL; 1.2% of women experienced drop >2 gm/dL
